# Hansbreen Snowpit Dataset – over 30-year of detailed snow research on an Arctic glacier

**DOI:** 10.1038/s41597-022-01767-8

**Published:** 2022-10-27

**Authors:** Michał Laska, Bartłomiej Luks, Daniel Kępski, Bogdan Gądek, Piotr Głowacki, Dariusz Puczko, Krzysztof Migała, Adam Nawrot, Michał Pętlicki

**Affiliations:** 1 Institute of Earth Sciences, Faculty of Natural Sciences, University of Silesia in Katowice, Będzinska 60, 41-200 Sosnowiec, Poland; 2 Institute of Geophysics, Polish Academy of Sciences, Księcia Janusza 64, 01-452 Warsaw, Poland; 3 Institute of Biochemistry and Biophysics Polish Academy of Sciences, Adolfa Pawińskiego 5a, 02-106 Warsaw, Poland; 4 Institute of Geography and Regional Development, University of Wrocław, Pl. Uniwersytecki 1, 50-137 Wrocław, Poland; 5 Faculty of Geography and Geology, Jagiellonian University in Kraków, Gronostajowa 7, 30-387 Kraków, Poland

**Keywords:** Cryospheric science, Databases

## Abstract

Snow cover is a key element in the water cycle, global heat balance and in the condition of glaciers. Characterised by high temporal and spatial variability, it is subject to short- and long-term changes in climatic conditions. This paper presents a unique dataset of snow measurements on Hansbreen, an Arctic glacier in Svalbard. The dataset includes 79 archived snow profiles performed from 1989 to 2021. It presents all available observations of physical properties for snow cover, such as grain shape and size, hardness, wetness, temperature and density, supplemented with organised metadata. All data has been revised and unified with current protocols and the present International Classification for Seasonal Snow on the Ground, allowing comparison of data from different periods and locations. The information included is essential for estimations of glacier mass balance or snow depth using indirect methods, such as ground-penetrating radar. A wide range of input data makes this dataset valuable to the greater community involved in the study of snow cover evolution and modelling related to glaciology, ecology and hydrology of glacierised areas.

## Abstract (summary)

**Table:** 

Measurement(s)	snow cover, snow stratigraphy
Technology Type(s)	snow pit analyses, automatic weather stations
Factor Type(s)	Hansbreen
Sample Characteristic - Environment	seasonal snow cover, cryosphere, polar environment
Sample Characteristic - Location	glacier, Hansbreen, Hornsund, Spitsbergen, Svalbard, Arctic

## Background & Summary

Detailed global snow cover analysis was initiated with a growing interest in photographic documentation of crystal forms, especially precipitation particles^1,2^. This became the basis for the first attempts to classify solid precipitation^3,4^, with subsequent attention paid to the physical processes occurring inside the snowpack – snow metamorphism^5,6^ – providing a complementary approach to classification^7–10^. In 2009, the International Association of Cryospheric Sciences (IACS) issued the present International Classification for Seasonal Snow on the Ground (ICSSG)^11^. It is based on the knowledge and achievements of snow researchers, who defined methods to determine the physical properties of snow cover around the world.

Svalbard is an archipelago located in the European sector of the Arctic, between 74° and 81°N. About 57% of its land area is covered by glaciers^12^, and due to its location, it is also one of the fastest-warming areas on Earth^13^. Detailed and systematic measurements of the physical characteristics of the snow cover on the glaciers of the southern part of Spitsbergen (the largest island of the archipelago) began in the late 1980s on the Hans Glacier (Hansbreen). This is a medium-sized tidewater glacier (54 km^2^) terminating into Hornsund Fiord, near (*c*. 3 km) to the Polish Polar Station Hornsund^14^. Snow measurements were begun as a part of a mass balance measurement program for this glacier.

Since 1989, the methodology applied to determining the stratigraphy of the seasonal snow cover has been changing. The main objective, and one of the most outstanding achievements of the work presented here, is the standardisation of collected data with the current ICSSG^11^. The standardisation of snow measurement protocols applied to this dataset is described in Table 1. In 1989–2004, snow research focused on the chemical properties of the snowpack, such as pH profiles, electrical conductivity, chloride ion concentration, and overall mineralisation^15,16^; consequently, much information concerning physical properties was not recorded. At that time, the physical characteristics of the snow cover were determined using the snow classification developed by Jan Leszkiewicz, Marian Pulina and Piotr Głowacki^15,17,18^, which was based on Kotlyakov’s classification^9^. It focused on grain shape and subjective assessment of size and hardness, without estimation of wetness. These classifications do not distinguish grain forms such as surface or depth hoar, however lack of those forms does not imply their absence at Hansbreen during that period. Where any doubts during the standardisation appeared, the layers were assigned to a double IACS code, where the first class described is primary and the next one is secondary. Additionally, no snowpit data has been found for 2001–2003 and 2005.

**Table 1:** The IACS codes^11^ for the snow classes by Colbeck^10^ and by Leszkiewicz and Pulina^17^, used in the descriptions of layering of the snowpack on the Hansbreen: PP – Precipitation Particles; DF – Decomposing and Fragmented precipitation particles; RG – Rounded Grains; FC – Faceted Crystals; DH – Depth Hoar; SH – Surface Hoar; MF – Melt Forms; MFcr – Melt-Freeze crust; IF – Ice Formations.

Snow class (symbol) by Leszkiewicz and Pulina^17^	IACS snow class code^11^	Snow class (symbol) by Colbeck *et al*.^10^	IACS snow class code^11^
fresh snow (1)	PP (DF)	precipitation particles (1)	PP
fine-grained snow, loose (2)	RG	decomposing and fragmented precipitation particles (2)	DF
medium-grained snow, loose (3)	RG	rounded grains (3)	RG
partly crystallised snow, loose (4)	RG (FC)	faceted crystals (4)	FC
medium and coarse-grained snow partly turned into firn (5)	FC (MF)	cup-shaped crystals; depth hoar (5)	DH
medium-grained firn (6)	MF	wet grains (6)	MF
coarse-grained firn (7)	MF	feathery crystals (7)	SH
glacier ice (8)	—	ice masses (8)	IF
firn ice (9)	—	melt-freeze crust (9e)	MFcr
damp snow (10)	MF		
wet snow (11)	MF		
slush (12)	MF		
ice layers with firned snow (13)	MFcr		
dense ice-crust (14)	MFcr		
compact ice-crust (15)	MFcr		
superimposed ice (16)	IF		
windblown snow (17)	DF (RG)		
deflated snow (18)	RG (FC)		
dense snow, not very compact (19)	RG (FC)		
compact snow (20)	RG		
very compact snow “gypsum” (21)	RG		
extremely compact snow “concrete” (22)	RG		

In 2006–2007, observers determined snow characteristics in the snowpits using the Leszkiewicz and Pulina classification^17^ and the classification introduced by Colbeck *et al*.^10^, which significantly improved the standardisation process over previous classifications. Measurements according to Colbeck’s protocol were carried out until 2013. Since 2014, when a transition to Fierz *et al*. classification^11^ began, the measurements conducted have been as complete as possible and follow the current ICSSG. According to the World Glacier Monitoring Service, since 2018, Hansbreen has become one of several reference glaciers, with more than 30 years of continuous glaciological mass-balance measurements^19^. Due to the COVID-19 pandemic, no snowpits were performed on Hansbreen in 2020. Nonetheless, the seasonal monitoring programme of the physical characteristics of snow cover is ongoing and will continue in the coming years.

The dataset presented here^20^ is the largest and the most prolonged compilation available of snowpit analyses across the Svalbard glaciers^21–24^. It includes all 79 available snow profiles collected on Hansbreen in 1989–2021. These were performed: to calibrate mass balance measurements and modelling;^19,25–31^ to estimate the snow cover depth using indirect methods, such as ground-penetrating radar (GPR)^32–34^ and remote sensing^35–37^; to determine the evolution of the seasonal snow cover^17,18,38–40^; and to calculate concentration and fluxes of pollutants deposited in the snowpack^23,41–44^.

**Fig. 1:**
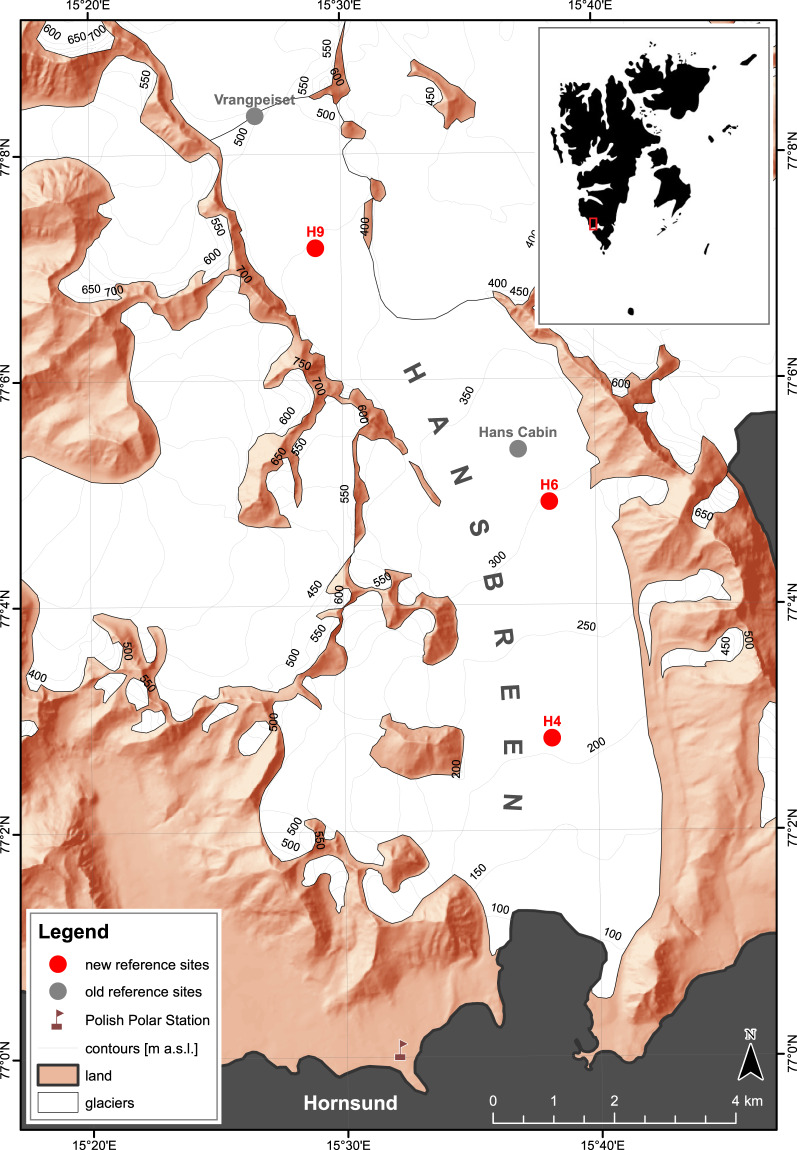
Study site with reference locations for the snowpit analyses: in 1989–2000 (grey dots, presenting locations in 1990) and in 2004–2021 (red dots, presenting locations in 2018). Source of vector data and Digital Elevation Model: Norwegian Polar Institute.

The snowpits were usually excavated once per season, in the period of maximum snow cover accumulation (April-May) at selected reference sites (Fig. 1). In the years 1989–2000, these were:

1. the glaciological station of the University of Silesia (Hans Cabin) area, later adjacent to the ablation stake no 6. Initially located at *c*. 320 m a.s.l., near to the equilibrium-line altitude (ELA); and
2. the Vrangpeiset, an ice divide between Hansbreen and Vrangpeisbreen, located at *c*. 500 m a.s.l. and represents the accumulation zone.

In the years 2004–2021, the reference sites were:

1. around ablation stake No. 4, located at *c*. 190 m a.s.l. (ablation zone);
2. around ablation stake No. 6, located at *c*. 290 m a.s.l. (ELA area);
3. around ablation stake No. 9, located at *c*. 420 m a.s.l. (accumulation zone).

**Fig. 2:**
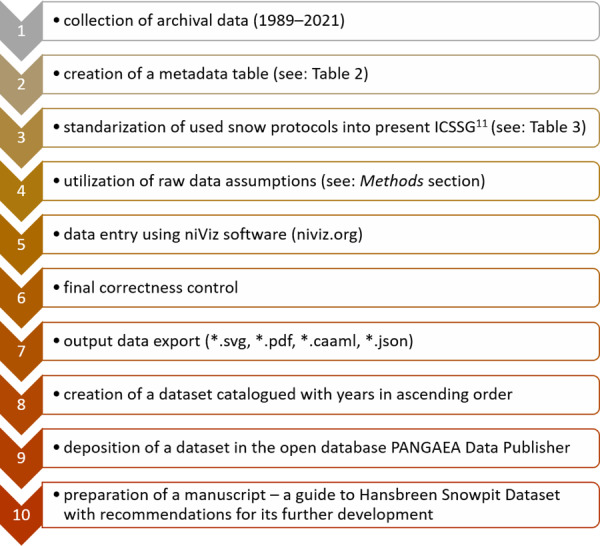
Flowchart of the data processing and creation of Hansbreen Snowpit Dataset.

Automatic Weather Stations (AWS) have been operated periodically at these sites. Their data were used to supplement the record of environmental conditions (air temperature, wind speed and direction) in the dataset metadata. The detailed workflow of these measurements is provided in Fig. 2.

The wide range of collected data makes the Hansbreen Snowpit Dataset^20^ valuable for researchers involved in the evolution and modelling of snow cover as related to glaciology, ecology or glacial hydrology. It can help provide information about the environmental background for the interdisciplinary research community planning to work on Hansbreen or act as a broad dataset for comparative analysis and discussion against other glacial systems in Svalbard or the entire Arctic exposed to rapid changes in climatic conditions.

## Methods

Over the past 30 years, many observers with varied snow sampling experience have been involved in determining the physical properties of snow cover. Therefore, it was necessary to adopt the following assumptions for the preparation, unification, and final elaboration of the data.

### Field measurements

The physical properties of seasonal snow cover presented in this study have been analysed from snowpits. Measurements included the determination of grain shape and size, snow hardness, wetness, density and temperature. Following international snow classifications^10,11^ since 2006, observers have applied the entire snowpit methodology, including simple hand tests, as have been thoroughly described there. Practical technical recommendations for snowpit measurements were combined in the Norsk Polarinstitutt report^45^.

### Dataset unification

**Fig. 3:**
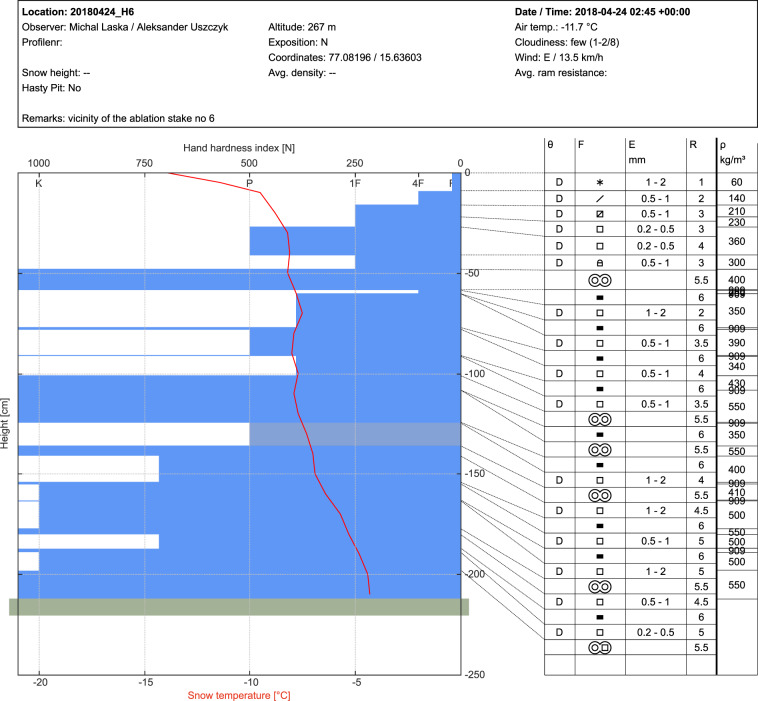
Example of the standardised snowpit data visualised with niViz software. The upper section presents metadata. The left section visualises snow layers by their hardness index. The right section describes detailed snow features: θ – wetness; F – grain shape; E – grain size [mean – maximum]; R – hardness; ρ – density. For the symbols and values explanation, see: ICSSG^11^.

For the development and visualisation of the entire dataset, the software *niViz* has been used. The technical details of this tool can be found in the *Usage Notes*. The effect of data standardisation is presented in Fig. 3 and clarified in the following text to support further development of the Hansbreen Snowpit Database in a unified form.

#### Name

The name of each profile has been coded as *YYYYMMDD_site location*.

#### Time and date

Described time refers to the moment when the snowpack analysis has begun. It is expressed in Coordinated Universal Time, UTC (±00: 00) for easier comparison with meteorological data from AWS on the glacier or data from Hornsund station (WIGOS Station Identifier 0-20000-0-01003). In the absence of a time recorded by the observer, the default time is arbitrarily set to 12:00 (±00:00).

#### Coordinates

Geographic coordinates and altitude of the snowpit positions in 2004–2021 were taken from direct GPS measurements or from the location of the nearby ablation stakes (dGPS survey). Where a lack of GPS measurements and/or signal scrambling due to Selective Availability before 2000 reduced accuracy by *c*. 100 m^46^, snowpit positions in 1989–2000 are instead based on high-resolution scans of the topographic map from 1990 with marked ablation stakes locations^47^, georeferenced in the UTM/ED50 system and then converted to UTM/WGS84 (EPSG: 32633). In general, the average uncertainty of the calculation is 0.3 m. Due to the glacier flow, which amounts to *c*. 0.085 m day^–1^ in the Hans Cabin area^48^, the locations provided should be treated as indicative.

#### Observers

The people mentioned as the observers measured the physical properties of the snow cover. The master observer is selected first. Those responsible for snow sampling for chemical analysis, as well as field assistants (e.g. note-takers, profile diggers) are not included.

#### Weather conditions

Meteorological conditions such as air temperature, wind speed and direction, sky condition, and precipitation are presented if recorded at the observation time only. The METAR standard (METeorological Aerodrome Report) for cloud cover and precipitation is used during data processing in dedicated software. A detailed code explanation is presented in the WMO Handbook^49^.

#### Snow grain shape and size

Grain shape, after Fierz *et al*.^11^, primarily described the main morphological classes, except single profiles, in which the observer used a detailed division into subclasses (2014, 2015, 2018, 2019). The only subclass, often specified even in the basic classification, was *Melt-freeze crusts (MFcr)*, distinguished due to their significant influence on melting dynamics^35,38^, circulation of water^50^, heat and matter transfer in the snow cover^51^, avalanche safety^52^ and relationship with *rain-on-snow* episodes^39^. Detailed information on unification from different classifications of grain shape is provided in the *Background* & *Summary* section and in Table 1. Grain size was measured on snow crystal cards with a 10x magnifier.

#### Snow density/hardness/wetness

The *Winter Engineering* snow density cutter, better known as the *Wasatch Touring 100*, is a cylindrical cutter with a volume of 100 cm^3^ ^53^. It was most often used to measure the density of snow layers. Alternatively, a box-shaped cutter with a digital scale was used in 2016 and Pesola spring scales (500 g and 1000 g) with a 100 cm^3^ cylinder cutter were used in 2012. Although simple cutters are light and handy for measuring the density of thin snow layers during fieldwork, they all have severe limitations in measuring dense, compact layers. Where possible, the density of hard layers was estimated based on the difference in bulk density measurements between the entire snow profile and individual layers. Such estimations assumed an *MFcr* density of 550 kg m^–3^. The fixed *IF* density was 909 kg m^–3^, after Watts *et al*.^54^. Detailed information on the density unification of density measurements from hard layers is included in the section *Technical Validation/Estimating the density of hard layers*. The hardness of *MFcr* was assumed to be 5.5 (knife to ice) and 6 (ice) for the *Ice Formations (IF)*. Wetness values were not defined for these layers.

#### Snow temperature

In 2006, 2007, and 2014−2018, the snow temperature was measured with an Elmetron PT-411 (uncertainty: ±0.9 °C; resolution 0.1 °C) and since 2019, with a Testo 110 combined with a penetration probe, thermocouple type K (uncertainty: ±0.2 °C; resolution 0.1 °C). Snow temperature (if recorded) was usually measured every 10 cm down the profile. Due to expected marked changes in temperature near the surface, it is recommended to measure the snow temperature on the snow surface (height = 0 cm), and then every 5 cm down to 20 cm below the surface^11,45^. In 2006, 2007 and 2016, the snow temperature was measured at irregular intervals in the midpoint of layers (where possible).

#### Snow chart X-axis

Snow classification^17^ used in 1989–2004 did not consider measuring such features as grain size, snow wetness or hardness because the research focused on chemical parameters of individual layers (pH, chloride ions, electrical conductivity, mineralisation) instead. For this reason, the primary parameter used in the snow profile visualisations is the density by the layer over the profile depth. The X-axis was also occasionally supplemented with snow temperature data in the following years.

#### Snow chart Y-axis

The zero level for all snow profiles follows the *zero-on-top* principle, describing subsequent profile layers from the youngest layer (the latest created) to the lowest, oldest layer (created at the beginning of the accumulation season). It is a base of the CAAML standard^55^ and common approach to analyse snow on glaciers in Svalbard^56–58^, Alaska^59^, Greenland^60^, Antarctica^61–63^, or Central Asia^64^., where a single annual profile analysis usually being done during the period of maximum accumulation. This is also the usual principle in chemical, biological, glaciological and black carbon/organic carbon studies^45^. Alternatively, mountain rescue services or snow research institutes (e.g. SLF-WSL) typically analyse their profiles from the oldest layer, *zero-on-bottom*, which is very beneficial with a defined ground level, and during analyses focused on the seasonal snow cover evolution, with regularly repeated measurements from the beginning of snow cover formation to complete disappearance. It also allows the implementation of data in models (e.g. SNOWPACK or CROCUS). Therefore *zero-on-bottom* methodology appears in avalanche research^65,66^, studies on snow evolution on the tundra^67,68^ and sea ice snowpack^69,70^ as well as in snow modelling^11,23,40^.

## Data Records

The dataset entitled: *Hansbreen Snowpit Dataset: a long-term snow monitoring (1989–2021) in the unique field laboratory (SW Spitsbergen, Svalbard)* is stored and accessible through the PANGAEA Data Publisher: 10.1594/PANGAEA.942279^20^.

A general overview of the Hansbreen Snowpit Dataset is presented in Table 2.

**Table 2:** General characteristics of the Hansbreen Snowpit Dataset.

Attribute	Value
Data type	Graphic and text script
Data format	SVG, PDF, CAAML, JSON
Spatial coverage	77.01–77.14°N; 15.44–15.61°E
Temporal coverage	27 March 1989–23 April 2021
Temporal resolution	Seasonal (spring)

Detailed information of the available and processed data (including metadata) can be found in Supplementary Table 1, summarising the entire dataset: 10.1038/s41597-022-01767-8.

## Technical Validation

The application of simple tests to measure the physical properties of snow cover is a challenge with respect to validation. Unless advanced technology with specialised scientific equipment is used, measurements may be fraught with significant errors and overdependence on the observer’s knowledge and experience. In this section, we will focus on density measurements, which were the motivation to start permanent monitoring of the seasonal snow cover whilst still providing critical information about the water equivalent of the snow cover, which is essential for the correct calculation of glacier mass balance.

### Estimating the density of hard layers

Until 2004, the snow density measurements referred only to the bulk density of the snowpack, as derived from samples obtained with a VS-43 snow core sampler, such as that used at the Polish Polar Station Hornsund. However, the structure of the snow cover on the glacier is much more complex than that on the tundra, and therefore has attracted more interest in detailed density measurements in vertical profiles. This is why small cylinder- and box-shaped cutters have been applied to measure snow layer density. A technical overview of snow density instrumentation is presented in Table 3. Comparisons of measurement methods have been already widely discussed^53,71–73^. Despite the advantage of measuring the density of layers of only several centimetres thickness, there is a severe limitation – sampling high hardness (hand hardness index >4.5) and high density (c. >450 kg m^–3^) layers is almost impracticable. This is an essential consideration because the contribution of such layers to snow profiles on Hansbreen can be in range of 6.4–36.5%^39^.

**Table 3:** Specification of density cutters used in the Hansbreen Snowpit Dataset.

Snow sampler	Type	Dimensions [mm]				Volume [cm^3^]
		Length	Width	Height	Diameter	
Winter Engineering/ Wasatch Touring 100	Cylinder	—	—	92	37.1	100
Box-shape cutter	Box	60	55	30	—	100
VS-43	Tube	—	—	600	79.8	3000

For *Ice Formations*, the density was fixed to 909 kg m^–3^ based on empirical studies by Watts *et al*.^54^. This value is within the range modeled by Wever *et al*.^74^, combining the observational data with the SNOWPACK model, while the physical ice density equals 917 kg m^–3^. The fixed density value for *Melt-Freeze crusts* assesses the underestimation of snow density by comparing bulk density from the VS-43 snow core sampler with the snowpack density from the *Winter Engineering* snow density cutter (excluding crust layers) in 2013–2015. This estimation was related to profiles located in different glacial zones and showed the most significant convergence at 550 kg m^–3^. It has been further validated using direct field measurements in 2016. The results show a mean density of the *Melt-Freeze Crusts* of 581 kg m^–3^ in the ablation zone (based on four layers), 560 kg m^–3^ in the ELA area (based on three layers) and 525 kg m^–3^ in the accumulation zone of Hansbreen (based on six layers). The decrease in measured values with elevation can be explained by the lower repeatability of the melt-freeze cycles in the colder, higher-located zones of a glacier. In this case, the mean measured *MFcr* density for the entire glacier equals 555 kg m^–3^. It is worth mentioning that the estimated value may be strongly affected by local conditions and should not be adopted for other locations without performing a similar calibration/validation. Although it is broadly in line with the literature^8,75^, much lower values can also be found^76,77^.

## Usage Notes

The data has been catalogued chronologically by year and contain in the description between one (e.g. 1991) and seven snow profiles (in 2010). In some years, measurements at the same sites were carried out two or even three times over intervals of several weeks. Apart from one profile in September 1989, analyses were conducted from March to June. Due to the shift in peak snow accumulation from April to mid-May, as observed over the past decade, a further increase in the number of snowpits excavated in May is probable.

The software *niViz* (https://niviz.org), version 0.9.4, was used to visualise the results^78^. It is an open-source software developed by the WSL Institute for Snow and Avalanche Research, SLF, based in Davos, Switzerland. *NiViz* is available under the GNU Affero General Public License (https://www.gnu.org). It is based on the IACS Snow Profiles international standard exchange format (http://caaml.org/Schemas/SnowProfileIACS/v6.0.3) that relies on ICSSG^11^. The software allows the user to enter, save, and export the data to both graphic (*.svg *.png) and text-based (*.caaml *.json) formats. Advanced users can build their own, customised version with the Git version control system. We recommend using this software for the archiving and later reuse of the data.

The authors’ assumption was to create and accurately describe the Hansbreen dataset and invite the snow research community to contribute to its further development. Use of the supplementary templates^79^ during snowpit analyses should allow the collection of all information required for their subsequent integration in*niViz*. These files are available at the Polish Polar Data Base https://ppdb.us.edu.pl/geonetwork/srv/eng/catalog.search#/metadata/c4596df9-bff7-41d8-8b6e-270df747a3cf and at Zenodo 10.5281/zenodo.6640776.

## Supplementary information

Supplementary Table 1

## Data Availability

No custom code has been created or used during the generation/unification and processing of this dataset.
