# Supplementary material for: Hansbreen Snowpit Dataset – over 30-year of detailed snow research on an Arctic glacier
Source: Sci Data. 2022 Oct 27;9:656. doi: 10.1038/s41597-022-01767-8 (PMC9610311; doi:10.1038/s41597-022-01767-8)
Supplement: Supplementary file 1 — Supplementary Table 1 [file 41597_2022_1767_MOESM1_ESM.pdf]

Year	Date (mm/dd)	Time [UTC]	Localization[A]	Coordinates[B]	Principal observer[C]	Classification type[D]	Snow depth [m]	Water equivalent of snow cover [mm]	Bulk density [kg m^{-3}]	Layer density[E]	Grain shape	Grain size	Hardness	Wetness	Temperature
2021	04/23	18:00	H4	F	BL	F	1.63	630.61	387	WE	✓	✓	✓	✓	✓
	04/23	15:00	H9	F	BL	F	2.55	935.70	367	WE	✓	✓	✓	✓	✓
2020															
2019	04/03	18:20	H6X	F	ML	F	1.56	592.84	380	WE	✓	✓	✓	✓	✓
	05/06	18:00	H9	F	BL	F	2.88	1110.4	386	WE	✓	✓	✓	✓	✓
2018	04/24	04:00	H4	S	ML	F	0.92	352.54	383	WE	✓	✓	✓	✓	✓
	04/24	02:45	H6	S	ML	F	2.12	863.27	407	WE	✓	✓	✓	✓	✓
	04/24	00:10	H9	S	ML	F	3.40	1352.36	398	WE	✓	✓	✓	✓	✓
2017	05/11	13:00	H4	F	BL	F	0.90	360.80	401	WE	✓	✓	✓	✓	✓
	05/12	16:00	H9	F	BL	F	3.07	1219.67	397	WE	✓	✓	✓	✓	✓
2016	04/25	09:00	H4	F	DK	F	1.02	394.11	386	BC	✓	✓	✓	✓	✓
	04/25	13:00	H6	F	DK	F	1.69	719.64	426	BC	✓	✓	✓	✓	✓
	04/29	12:10	H9	F	DK	F	2.88	1296.48	450	BC	✓	✓	✓	✓	✓
2015	04/29	11:30	H4	F	ML	F	1.45	710.10	490	WE	✓	✓	✓	✓	✓
	04/29	18:00	H6	F	ML	F	2.00	890.81	445	WE	✓	✓	✓	✓	✓
	04/29	13:00	H9	F	ML	F	3.00	1319.90	440	WE	✓	✓	✓	✓	✓
	05/23	16:10	H4	F	ML	F	1.65	786.56	477	WE	✓	✓	✓	✓	✓
	05/23	12:20	H6	F	ML	F	2.20	1022.93	465	WE	✓	✓	✓	✓	✓
2014	05/05	16:00	H4	F	ML	F	1.60	592.92	371	WE	✓	✓	✓	✓	✓
	05/05	20:00	H6	F	ML	F	2.10	828.50	395	WE	✓	✓	✓	✓	✓
	05/06	01:00	H9	F	ML	F	2.78	1199.06	431	WE	✓	✓	✓	✓	✓
	06/13	13:00	H4	F	DK	F	1.02	494.99	485	WE	✓	✓	✓	✓	✓
2013	05/11	20:00	H6	F	ML	C	1.72	769.36	447	WE	✓	✓	✓	✓	×
	05/11	17:00	H9	F	ML	C	3.15	1431.52	454	WE	✓	✓	✓	✓	×
	05/11	23:40	H4	F	ML	C	1.20	526.91	439	WE	✓	✓	✓	✓	×
2012	05/12	17:00	H9	S	AN	C	4.45	1879.88	413	P	✓	✓	✓	✓	×
	05/21	12:10	H4	F	AN	C	1.98	898.70	454	P	✓	✓	✓	✓	×
	05/21	×	H6	S	AN	C	2.03	918.99	453	P	✓	✓	✓	✓	×
	06/21	10:00	H4	S	ML	C	1.33	672.60	506	WE	✓	✓	✓	✓	×
	06/21	12:45	H6	S	ML	C	1.54	872.05	566	WE	✓	✓	✓	✓	×

2011	04/16	20:00	H4	S	DP	C	1.72	743.40	432	WE	✓	✓	✓	✓	×
	04/16	13:00	H9	S	DP	C	4.17	1830.68	439	WE	✓	✓	✓	✓	×
2010	04/18	10:00	H4	F	DP	C	1.57	658.30	419	WE	✓	✓	✓	✓	×
	04/19	15:30	H9	F	DP	C	4.53	1929.81	426	WE	✓	✓	✓	✓	×
	04/21	14:00	H6	F	ML	C	2.41	1073.52	445	WE	✓	✓	✓	✓	×
	05/13	12:30	H4	F	ML	C	1.68	799.18	476	WE	✓	✓	✓	✓	×
	05/20	10:45	H6	F	ML	C	2.33	1123.47	482	WE	✓	✓	✓	✓	×
	05/25	15:25	H6	F	ML	C	2.00	1108.40	554	WE	✓	✓	✓	✓	×
	05/30	10:50	H4	F	ML	C	0.55	286.19	520	WE	✓	✓	✓	✓	×
2009	04/24	×	H4	S	DP	C	1.63	×	×	×	✓	✓	✓	✓	×
	05/07	×	H9	S	DP	C	3.00	×	×	×	✓	✓	✓	✓	×
2008	04/21	×	H1	S	DP	C	1.20	598.49	499	WE	✓	✓	✓	✓	×
	04/26	15:00	H4	S	DP	C	2.40	1109.00	462	WE	✓	✓	✓	✓	×
	04/19	19:00	H6	S	DP	C	2.72	1168.20	429	WE	✓	✓	✓	✓	×
	04/24	00:10	H9	S	DP	C	3.98	1710.54	430	WE	✓	✓	✓	✓	×
2007	04/20	×	H2	S	AN	LP/C	1.52	549.60	362	WE	✓	✓	✓	✓	✓
	04/16	×	H4	S	AN	LP/C	2.20	940.40	427	WE	✓	✓	✓	✓	✓
	04/19	×	H9	S	AN	LP/C	3.12	1355.09	434	WE	✓	✓	✓	✓	✓
2006	04/12	×	H1	×	DP	LP/C	1.43	577.26	404	WE	✓	✓	✓	✓	✓
	04/10	×	H2-3	×	DP	LP/C	1.68	750.26	447	WE	✓	✓	✓	✓	✓
	04/24	×	H4	×	KM	LP/C	2.90	1247.45	430	WE	✓	✓	✓	✓	✓
	04/26	×	H9	×	DP	LP/C	5.75	2539.43	442	WE	✓	✓	✓	✓	✓
2005															
2004	05/10	10:00	H4	×	KM	LP	2.30	1085.23	472	×	✓	×	×	×	×
2003															
2002															
2001															
2000	05/22	×	H1	M	×	LP	1.17	495.58	424	×	✓	×	×	×	×
	05/16	×	H2	M	×	LP	2.45	1172.35	479	×	✓	×	×	×	×
	05/16	×	H6	M	×	LP	1.55	644.00	415	×	✓	×	×	×	×
	05/15	×	H10	M	×	LP	3.43	1541.80	450	×	✓	×	×	×	×
1999	05/03	×	Hf	M	MP	LP	1.07	383.90	359	×	✓	×	×	×	×
	04/19	×	HC	M	MP	LP	1.80	706.00	392	×	✓	×	×	×	×
	04/22	×	Vp	×	MP	LP	2.46	972.30	395	×	✓	×	×	×	×
1998	05/30	×	HC	M	PG	LP	2.23	911.06	409	×	✓	×	×	×	×
1997	05/02	×	HC	M	PG	×	2.15	865.65	403	×	×	×	×	×	×
1996	04/15	×	HC	M	PG	LP	2.13	822.40	386	×	✓	×	×	×	×

1995	05/31	×	HC	M	PG	×	1.45	639.20	441	×	×	×	×	×	×
	06/02	×	Vp	×	PG	×	1.85	760.10	411	×	×	×	×	×	×
	06/28	×	Vp	×	PG	×	1.50	760.80	507	×	×	×	×	×	×
1994	04/20	×	HC	M	PG	×	1.80	729.90	406	×	×	×	×	×	×
	04/23	×	Vp	×	PG	×	1.90	706.70	372	×	×	×	×	×	×
1993	04/15	12:20	HC	M	JL	LP	2.05	747.90	365	×	✓	×	×	×	×
	05/21	18:00	Vp	×	JL	LP	2.15	841.10	391	×	✓	×	×	×	✓
1992	04/21	×	HC	M	MP	LP	1.65	599.50	363	×	✓	×	×	×	×
	04/14	×	Vp	×	MP	LP	2.20	917.10	417	×	✓	×	×	×	×
1991	05/03	×	HC	M	PG	LP	3.10	1377.20	444	×	✓	×	×	×	×
1990	04/07	×	HC	M	MP	×	2.05	896.50	437	×	×	×	×	×	×
	04/05	×	Vp	×	MP	×	2.50	962.00	385	×	×	×	×	×	×
1989	03/27	10:40	Hf	×	JL	LP	1.06	×	×	×	✓	×	×	×	×
	03/30	12:10	HC	M	JL	LP	1.66	552.20	333	×	✓	×	×	×	×
	04/11	×	HC	M	PG	×	1.92	641.70	334	×	×	×	×	×	×
	05/01	19:30	Vp	×	JL	LP	2.12	783.90	370	×	✓	×	×	×	×
	09/03	×	J4	×	PG	LP	1.58	745.64	472	×	✓	×	×	×	×

Supplementary Table 1. Summary presenting available data in Hansbreen Snowpit Dataset. Data: available (blue), not measured (red), no data (black). Abbreviations: [A]Localization: H[number] – the position of a nearby ablation stake, HC – Hans Cabin, Hf – Hansbreen front, Vp – Vrangpeiset; [B]Coordinates: F – fixed GPS location, M – estimation based on map calibration, S – estimation based on ablation stake location; [C]Principal observer: AN – Adam Nawrot, BL – Bartłomiej Luks, DK – Daniel Kępski, DP – Dariusz Puczko, JL – Jan Leszkiewicz, KM – Krzysztof Migała, ML – Michał Laska, MP – Marian Pulina, PG – Piotr Głowacki; [D]Classification type: F – Fierz et al.[11], C – Colbeck et al.[10], LP – Leszkiewicz and Pulina[17]; [E]Layer density: WE – Winter Engineering snow density cutter, BC – box-shaped density cutter with a digital scale, P – Pesola spring scale with a 100 cm^3 cylinder cutter. For the complete reference, see: the manuscript.
